# Improving gene set analysis of microarray data by SAM-GS

**DOI:** 10.1186/1471-2105-8-242

**Published:** 2007-07-05

**Authors:** Irina Dinu, John D Potter, Thomas Mueller, Qi Liu, Adeniyi J Adewale, Gian S Jhangri, Gunilla Einecke, Konrad S Famulski, Philip Halloran, Yutaka Yasui

**Affiliations:** 1 Department of Public Health Sciences, School of Public Health, University of Alberta, Edmonton, Alberta, T6G 2G3, Canada; 2 Division of Public Health Sciences, Fred Hutchinson Cancer Research Center, Seattle, Washington, 98109-1024, USA; 3 Division of Nephrology & Transplantation Immunology, Faculty of Medicine and Dentistry, University of Alberta, Edmonton, Alberta, T6G 2S2, Canada

## Abstract

**Background:**

*Gene-set *analysis evaluates the expression of biological pathways, or *a priori *defined gene sets, rather than that of individual genes, in association with a binary phenotype, and is of great biologic interest in many DNA microarray studies. Gene Set Enrichment Analysis (GSEA) has been applied widely as a tool for gene-set analyses. We describe here some critical problems with GSEA and propose an alternative method by extending the individual-gene analysis method, Significance Analysis of Microarray (SAM), to gene-set analyses (SAM-GS).

**Results:**

Using a mouse microarray dataset with simulated gene sets, we illustrate that GSEA gives statistical significance to gene sets that have no gene associated with the phenotype (null gene sets), and has very low power to detect gene sets in which half the genes are moderately or strongly associated with the phenotype (truly-associated gene sets). SAM-GS, on the other hand, performs very well. The two methods are also compared in the analyses of three real microarray datasets and relevant pathways, the diverging results of which clearly show advantages of SAM-GS over GSEA, both statistically and biologically. In a microarray study for identifying biological pathways whose gene expressions are associated with *p53 *mutation in cancer cell lines, we found biologically relevant performance differences between the two methods. Specifically, there are 31 additional pathways identified as significant by SAM-GS over GSEA, that are associated with the presence vs. absence of *p53*. Of the 31 gene sets, 11 actually involve *p53 *directly as a member. A further 6 gene sets directly involve the extrinsic and intrinsic apoptosis pathways, 3 involve the cell-cycle machinery, and 3 involve cytokines and/or JAK/STAT signaling. Each of these 12 gene sets, then, is in a direct, well-established relationship with aspects of *p53 *signaling. Of the remaining 8 gene sets, 6 have plausible, if less well established, links with *p53*.

**Conclusion:**

We conclude that GSEA has important limitations as a gene-set analysis approach for microarray experiments for identifying biological pathways associated with a binary phenotype. As an alternative statistically-sound method, we propose SAM-GS. A free Excel Add-In for performing SAM-GS is available for public use.

## Background

Some DNA microarray studies may target discovery of individual genes whose expressions are associated with a phenotype. Useful statistical approaches have been proposed for such individual-gene analyses, for example, Significance Analysis of Microarray (SAM) in [1]. In many instances, however, the goal of studies is in the assessment of biologic pathways, or *a priori *defined gene sets, in association with a phenotype, i.e., gene-set analyses. Computationally, gene-set analyses require an additional consideration over individual-gene analyses, namely, the incorporation of gene sets into an association measure. Mootha et al. [2] proposed Gene Set Enrichment Analysis (GSEA) for gene-set analysis, utilizing the Kolmogorov-Smirnov statistic to measure the degree of differential gene expression in a gene set across binary phenotypes. GSEA was revised in 2005 by the same research team, replacing the Kolmogorov-Smirnov statistic with its weighted version to avoid certain deficiencies in the original GSEA method [3]. Many methods have been proposed since for gene-set analyses: see a recent excellent review by Goeman and Bühlmann [4] and their criticisms on the majority of the existing methods. Among the work not covered by the review by Goeman and Bühlmann, Tian et al. [5], in particular, made an important contribution by distinguishing different types of hypotheses in gene-set analysis, and proposed a permutation-based inference using the sum of t-statistic across genes in the gene set as a test statistic. In spite of the large number of gene-set analysis methods, however, GSEA remains by far the most widely used gene-set analysis method to date.

We propose here an alternative, an extension of SAM, to gene-set analysis, called hereafter SAM-GS. This is motivated by our observation that GSEA, in both the original and revised versions, fails to satisfy certain required properties that a gene-set analysis method should satisfy: for example, a gene-set analysis should not indicate an association for a gene set in which no gene is associated with the phenotype. In this paper, we first illustrate the behavior of GSEA in relation to a few simple, required properties of a gene-set analysis method and compare it with the behavior of SAM-GS, using a mouse-microarray kidney-transplant dataset. We then re-analyze, by SAM-GS, three DNA microarray datasets with which the application of GSEA was illustrated in [3], showing appreciable differences in the analysis results. The differences of the results are discussed from both biologic and statistical points of view, pointing out clear advantages of SAM-GS over GSEA.

## Results and discussion

### Gene-set simulation experiment

Using a mouse-microarray kidney-transplant dataset, we assessed if GSEA and SAM-GS satisfy the following simple requisite properties for any methods designed to perform a gene-set analysis:

(a) If the gene set *S *consists of genes whose expressions are consistently not associated with the phenotype *D*, the method should not indicate that *S *is associated with *D*.

(b) If the gene set *S *consists of a mix of genes with moderate to strong and weak associations of expressions with the phenotype *D*, such that an appreciable subset of the genes in *S *are moderately or strongly associated with the phenotype *D*, the method should indicate that *S *is associated with *D*.

(c) The size of the gene set *S *should not greatly alter the statistical significance in (a) and (b).

We performed two tests using the mouse-microarray kidney-transplant dataset with simulated gene sets. For Test 1 intended to assess property (a), the gene sets were randomly generated from the null hypothesis region: the expression of genes in these null gene sets were not associated with the phenotype. A sensible gene-set analysis method should not identify these gene sets as statistically significant. For Test 2 intended to assess property (b), the gene sets were generated randomly from an alternative hypothesis region: the expressions of 50% of genes in each gene set were associated with the phenotype either moderately or strongly. A sensible gene-set analysis method should identify these gene sets as statistically significant.

Tables 1 and 2 show the percentages of 100 randomly-generated gene sets whose expressions were found to be associated with the phenotype with a p-value ≤ 0.05, by the gene-set analysis method of interest (GSEA or SAM-GS), under each gene-set sampling region considered. In Test 1, null gene sets were generated by randomly sampling genes from genes with |*r*| <*c*, where *r *denotes the Pearson correlation coefficient of the gene expression with the phenotype and *c *was 0.01, 0.02, 0.05, 0.1, 0.2, 0.3, 0.4, or 0.5. The results in Table 1 indicate that GSEA does not satisfy requisite property (a), because it identified many of these null gene sets to be associated with the phenotype with a p-value ≤ 0.05. In Test 2, truly-associated gene sets were generated by randomly sampling half of the gene set's genes from genes with |*r*| = *c *and remaining half from genes with |*r*| <*c*, where *c *was 0.4, 0.5, 0.6, 0.7, 0.8, or 0.9. The results in Table 2 indicate that GSEA does not satisfy requisite property (b), because it did not identify many of these truly-associated gene sets as statistically significant with a p-value ≤ 0.05. Moreover, GSEA does not satisfy property (c), as the performance of the method varies greatly with gene-set size.

**Table 1:** Performance of GSEA and SAM-GS on Test 1. Proportions of randomly generated null gene sets that are identified by each method to be associated with the phenotype (p-value ≤ 0.05) in a mouse-microarray study.

**Correlation range from which gene-set members were selected (% of individual genes in the range) [% of individual genes in the range with FDR ≤ 0.01]**	**Methods**	**Set Size**			
		
		**10**	**30**	**50**	**100**
|*r*| < 0.01 (.9% of all genes are in the range) [0% with FDR≤0.01]	GSEA	100%	100%	100%	100%
	
	SAM-GS	0%	0%	0%	0%

|*r*| < 0.02 (2% of all genes are in the range) [0% with FDR≤0.01]	GSEA	100%	100%	100%	100%
	
	SAM-GS	0%	0%	0%	0%

|*r*| < 0.05 (4% of all genes are in the range) [0% with FDR≤0.01]	GSEA	100%	100%	100%	100%
	
	SAM-GS	0%	0%	0%	0%

|*r*| < 0.1 (8% of all genes are in the range) [0% with FDR≤0.01]	GSEA	100%	100%	100%	100%
	
	SAM-GS	0%	0%	0%	0%

|*r*| < .2 (16% of all genes are in the range) [0% with FDR≤0.01]	GSEA	96%	100%	100%	100%
	
	SAM-GS	0%	0%	0%	0%

|*r*| < .3 (25% of all genes are in the range) [0% with FDR≤.01]	GSEA	13%	100%	100%	100%
	
	SAM-GS	0%	0%	0%	0%

|*r*| < .4 (36% of all genes are in the range) [0% with FDR≤.01]	GSEA	8%	100%	100%	100%
	
	SAM-GS	0%	0%	0%	0%

|*r*| < .5 (47% of all genes are in the range) [0% with FDR≤.01]	GSEA	0%	24%	100%	100%
	
	SAM-GS	0%	0%	0%	0%

**Table 2:** Performance of GSEA and SAM-GS on Test 2. Proportions of randomly generated non-null gene sets that are identified by each method to be associated with the phenotype (p-value ≤ 0.05) in a mouse-microarray study.

**Pearson correlation of genes in the gene set with the phenotype**	**Methods**	**Set Size**			
		
		**10**	**30**	**50**	**100**
Half of genes with |*r*| ≥ .4, the other half with |*r*| < .4	GSEA	1%	3%	0%	1%
		
	SAM-GS	93%	100%	100%	100%

Half of genes with |*r*| ≥ .5 the other half with |*r*| < .5	GSEA	3%	4%	3%	1%
		
	SAM-GS	100%	100%	100%	100%

Half of genes with |*r*| ≥ .6, the other half with |*r*| < .6	GSEA	6%	7%	7%	18%
		
	SAM-GS	100%	100%	100%	100%

Half of genes with |*r*| ≥ .7 the other half with |*r*| < .7	GSEA	12%	18%	31%	66%
		
	SAM-GS	100%	100%	100%	100%

Half of genes with |*r*| ≥ .8, the other half with |*r*| < .8	GSEA	20%	64%	88%	100%
		
	SAM-GS	100%	100%	100%	100%

Half of genes with |*r*| ≥ .9, the other half with |*r*| ≥ .9	GSEA	69%	100%	100%	100%
		
	SAM-GS	100%	100%	100%	100%

**Figure 1:**
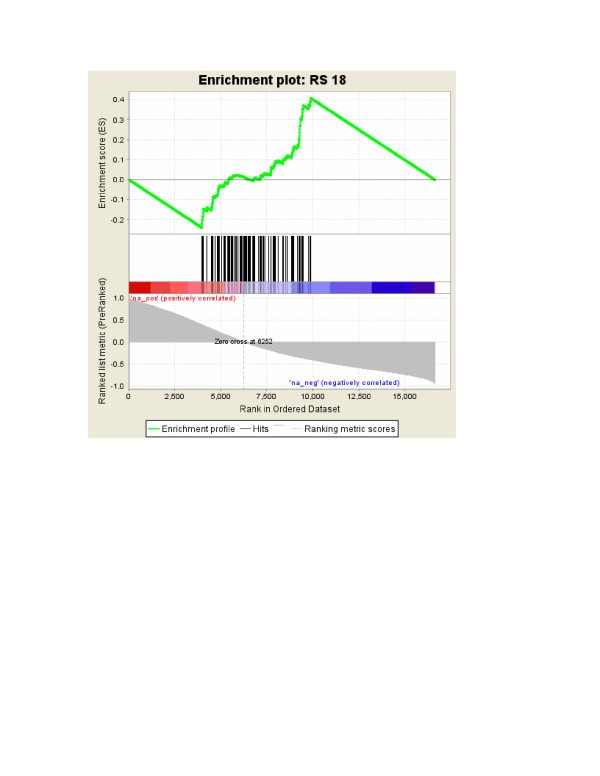
A statistically significant GSEA result. An illustration of a statistically-significant GSEA result with 100 genes selected at random from genes with no or weak correlation of expression with the phenotype (|*r*| < 0.4).

The results of these tests illustrate two situations where GSEA fails. One is where genes in a gene set cluster somewhere other than in the strong-association region (e.g., all individual genes could have no or very weak association with the phenotype) and GSEA identifies the gene set to be statistically significantly associated with the phenotype. Figure 1 illustrates this situation with one of the null gene sets of Test 1, where the gene set's genes clustered in the region of no or weak association with the phenotype, and yet GSEA p-value of this gene set was < 0.001. In short, GSEA will indicate that gene sets with any clear clustering are statistically significant, regardless of where the clustering occurs. The other situation is where a gene set has a mixture of moderately or strongly associated genes and weakly associated genes. This mixture within a gene set seems biologically plausible: not all genes in a phenotype-associated pathway will show changes in relation to the phenotype. GSEA has very poor power for detecting a differentially-expressed gene set under such mixed situations (Table 2), unless the clustering of some of the gene-set members occur at the moderate-strong association region (e.g., Table 2 with *c *= 0.9)

To check whether SAM-GS satisfies the three requisite properties of a gene-set analysis, the same tests were applied as for GSEA, using the same randomly-sampled simulated gene sets. The results of Tests 1 and 2 displayed in Tables 1 and 2 indicate that SAM-GS satisfies properties (a) and (b). Moreover, SAM-GS satisfies property (c), as its performance did not vary with the size of the gene set.

### Gene-set analyses of the three datasets with biologically defined gene sets

We compared the performance of the two methods, GSEA and SAM-GS, on the analyses of biologically defined gene sets using three microarray datasets considered in [3]: the sex, *p53*, and leukemia datasets. The sex dataset consists of mRNA expression profiles from lymphoblastoid cell lines derived from 15 males and 17 females (the phenotype is sex). The *p53 *dataset consists of expressions for 50 cell lines from the NCI-60 collection of cancer cell lines, for which mutational status of the *p53 *gene has been reported, with 17 being classified as wild-type, and 33 as carrying mutations in the gene (the phenotype is the mutation status of *p53*). The leukemia dataset consists of gene expression profiles of cells from 24 acute lymphoid leukemia (ALL) patients and 24 acute myeloid leukemia (AML) patients (the phenotype is ALL vs. AML). The pathways are organized in two catalogs, C1 and C2. The C1 catalog includes gene sets corresponding to human chromosomes and cytogenetic bands, while the C2 catalog includes gene sets that are involved in specific metabolic signaling pathways [3]. The results by GSEA and SAM-GS are summarized in Table 3. In the sex-comparison analysis, the two methods agreed on the associations (FDR ≤ 0.01) with the three Y associated gene sets, the testis-expressed gene set (GSEA FDR = 0.02), and the gene set with genes that escape X inactivation. In addition, SAM-GS established an association with the chrXp22 gene set (SAM-GS FDR ≤ 0.16 vs. GSEA FDR = 1.00). In the *p53*-comparison analysis, SAM-GS and GSEA agreed on a subset of gene sets with an FDR ≤ 0.01 that included the gene sets of hsp27, *p53*_UP (GSEA FDR = 0.013), *p53 *hypoxia, radiation sensitivity (GSEA FDR = 0.07), and *p53 *(BioCarta). However, SAM-GS identified additional 31 gene sets with an FDR ≤ 0.01, all of which had an FDR ≥ 0.49 for GSEA. These gene sets are shown in Table 4. In the leukemia-comparison dataset, the two methods gave even more discrepant results than the *p53*-comparison analysis. GSEA identified only five gene sets with an FDR ≤ 0.25 (none with an FDR ≤ 0.01), whereas all of the 182 gene sets were statistically significant (FDR ≤ 0.01) by SAM-GS. Note that the individual-gene analysis showed that 80% of the individual genes in this comparison had an FDR ≤ 0.25, which is in line with the gene-set analysis results of SAM-GS.

**Table 3:** Results of the analyses of three datasets by GSEA and SAM-GS.

**Dataset**	% of individual genes with FDR* ≤ 0.25	**# of gene sets with FDR ≤ 0.01**		**# of gene sets with FDR ≤ 0.25**		**Sensitivity/Specificity (AUC^†^) of GSEA**^‡^
			
		**GSEA**	**SAM-GS**	**GSEA**	**SAM-GS**	
**Sex**	0.1%	4	5	6	6	0.78/0.98 (0.94)
***p53***	0.3%	3	36	6	308	0.21/0.94 (0.68)
**Leukemia**	79.9%	0	182	5	182	0.06/NA^§ ^(NA^§^)

* FDR = False discovery rate estimate

^† ^AUC = Area under the ROC curve

^‡ ^Taking SAM-GS p ≤ 0.05 as the target to be predicted

^§ ^All gene sets in the leukemia dataset had SAM-GS p ≥ 0.05

**Table 4:** The 31 gene sets for which SAM-GS and GSEA strongly disagreed (SAM-GS FDR ≤ 0.01, GSEA FDR ≥ 0.49) in the *p53 *analysis.

**Gene Set**	**GSEA**		**SAM-GS**		***p53 *link**
	**FDR**	**p-value**	**FDR**	**p-value**	

**ATM Pathway**	0.87	0.21	≤ 0.01	< 0.001	Pathway member
**BAD Pathway**	0.57	0.04	≤ 0.01	< 0.001	Apoptosis
**Calcineurin Pathway**	0.84	0.13	≤ 0.01	< 0.001	*p53*-induced proline oxidase mediates apoptosis via a calcineurin-dependent pathway (12)
**Cell cycle regulator**	0.90	0.29	≤ 0.01	< 0.001	Cell cycle
**Mitochondria pathway**	0.88	0.32	≤ 0.01	< 0.001	Apoptosis
***p53 *signaling pathway**	0.51	0.01	≤ 0.01	< 0.001	Pathway member
**Raccycd Pathway**	0.83	0.56	≤ 0.01	< 0.001	Cell cycle
**SA_TRKA_RECEPTOR**	0.83	0.34	≤ 0.01	< 0.001	Integrated negative feedback loop between Akt and *p53 *(11)
**bcl2family and reg. network**	0.83	0.42	≤ 0.01	0.001	Apoptosis
**Cell cycle arrest**	0.98	0.49	≤ 0.01	0.001	Cell cycle
**Ceramide Pathway**	0.88	0.30	≤ 0.01	0.001	Apoptosis
**DNA DAMAGE SIGNALLING**	0.85	0.23	≤ 0.01	0.002	Pathway member
**SIG_IL4RECEPTOR IN_B_LYMPHOCYTES**	0.93	0.27	≤ 0.01	0.002	Cytokines; JAK/STAT signaling
**Cell cycle Pathway**	0.89	0.72	≤ 0.01	0.003	Pathway member
**G2 Pathway**	0.81	0.50	≤ 0.01	0.003	Pathway member
**Chemical Pathway**	0.53	0.04	≤ 0.01	0.005	Pathway member
**Drug resistance and metabolism**	0.86	0.08	≤ 0.01	0.005	Pathway member
**G1 Pathway**	0.81	0.37	≤ 0.01	0.005	Pathway member
**Breast cancer estrogen signaling**	1.00	0.85	≤ 0.01	0.006	Pathway member
**Ca_nf_at_signaling**	0.78	0.08	≤ 0.01	0.007	Apoptosis (and cytokines)
**Cytokine Pathway**	0.53	0.05	≤ 0.01	0.007	Cytokines
**ST_Interleukin_4_Pathway**	0.84	0.07	≤ 0.01	0.007	Cytokines; JAK/STAT signaling
**CR_DEATH**	0.86	0.31	≤ 0.01	0.008	Pathway member
**MAP00860: Porphyrin & chlorophyll metabolism**	0.92	0.29	≤ 0.01	0.010	CPO regulated by *p53 *(13)
**Ck1 Pathway**	0.49	0.02	≤ 0.01	0.011	Cdk5 phosphorylates *p53 *(9)
**Hivnef Pathway**	0.95	0.48	≤ 0.01	0.011	Apoptosis
**Ets Pathway**	0.79	0.45	≤ 0.01	0.012	Ets1 required for *p53 *transcriptional activation in UV-induced apoptosis (10)
**ST_Wnt_Ca2_cyclic_GMP_Pathway**	0.80	0.13	≤ 0.01	0.012	At least one known link between wnt and *p53 *(14)
**Chrebp Pathway**	0.84	0.42	≤ 0.01	0.013	unknown
**GPCRs_Class_A_Rhodopsin-like**	0.60	0.04	≤ 0.01	0.013	unknown
**ST_Fas_Signaling_Pathway**	0.80	0.52	≤ 0.01	0.013	Pathway member

These discrepancies between the two methods are summarized along with the sensitivity and specificity of the GSEA p-value ≤ 0.05 and the area under the receiver operating characteristic curve of GSEA p-value in predicting the SAM-GS p-value ≤ 0.05 (Table 3). Specifically, sensitivity was calculated as the proportion of gene sets with GSEA p-values ≤ 0.05, out of all gene sets with SAM-GS p-values ≤ 0.05. Specificity was calculated as the proportion of gene sets with GSEA p-values > 0.05, out of all gene sets with SAM-GS p-values > 0.05.

### GSEA

Our Tests 1 and 2 suggest that GSEA does not meet some simple requisite criteria for a gene-set analysis method. In particular, Test 1 results suggest that, in a typical microarray experiment involving genes with different degrees of association with the phenotype, GSEA would frequently identify gene sets as statistically significant when all of its genes have observed expressions completely uncorrelated with the phenotype (e.g., Pearson correlation between -0.1 and 0.1). This is not a logical behavior for a gene-set analysis method. Biologically, if a gene set is identified as having expressions that are significantly associated with a phenotype, the gene set should contain at least some genes whose observed expressions are associated with the phenotype. Statistically, the false discovery rate of GSEA for a truly null gene set, tested among truly non-null gene sets, would be appreciably elevated because the observed correlations of the null-gene-set genes with the phenotype would tend to cluster near zero. Although the gene sets in Tests 1 and 2 are randomly-sampled simulated sets, they are not unrealistic gene sets. For example, a Test 1 situation was encountered in the analysis of the sex dataset, where GSEA gave the "cell-cycle arrest genes" a p-value of 0.015 in association with sex (SAM-GS p-value = 0.84). No gene in this gene set has an absolute value of the Pearson correlation of 0.33 or greater, or the SAM p-value < 0.06: this clustering is thus identified incorrectly by GSEA as showing a significant association, failing Test 1. A Test 2 situation was encountered, for example, in the analysis of the leukemia dataset, where GSEA failed to identify the gene set "chr10q24", even though 13 of the 43 genes in the gene set had absolute values of the Pearson correlation of 0.5 or greater (4 genes greater than 0.7) and the chromosomal location of the gene set is biologically relevant given the role of *HOX11 *in T-cell ALL. The use of GSEA is subject to appreciable false positive and negative findings, as illustrated by the two tests and the results shown in Table 4.

Another critical problem of GSEA is its *relative *ranking of genes in a gene set in relation to the other genes outside of the gene set. The use of a relative measure in GSEA, rather than an absolute measure, means that important information on the degree of association between each gene and the binary phenotype is discarded. For example, the leukemia dataset had 80% of its 10,056 individual genes with an FDR ≤ 0.25. Regardless of whether such clear differences in gene expression across the binary phenotype are determined by biology, or by more mundane (and biologically irrelevant) differences in sample collection or handling, a gene-set analysis of this dataset should find that many gene sets are associated with the phenotype. GSEA, however, found only five gene sets with an FDR ≤ 0.25 in the leukemia-comparison analysis, inconsistent with the individual-gene analysis results. The cause of the inconsistency is the use of the relative ranking in GSEA. In contrast, SAM-GS found all gene sets in the leukemia dataset to have an FDR ≤ 0.01.

A related, perhaps less serious, issue with GSEA is that, when an individual gene set is of biologic interest, the SAM-GS analysis requires measurement only of the expression of the genes in the gene set to construct the test statistic (except the calculation of *s*_0_), whereas GSEA requires measurement of the expression of all genes to provide a relative ranking of all genes. The expression levels of the other genes should not affect the inference on an individual gene set of interest, if the individual set is, indeed, the only biologically relevant variable.

Another problematic aspect of GSEA is that its enrichment score considers genes with positive and negative associations with the phenotype separately, even when they have the same degree of associations with the phenotype. Thus, a gene set with a mix of genes with positive and negative associations with the phenotype, although biologically plausible (for instance, due to feedback loops in pathways), is not appropriately evaluated for association with the phenotype by the enrichment score and, therefore, has an improperly low probability of being detected as a phenotype-associated gene set by GSEA.

A gene-set analysis utilizes existing biologic knowledge that maps individual genes into gene sets or pathways. Because of the utilization of existing knowledge in the analysis, a well conducted gene-set analysis can be remarkably powerful. The *p53 *analysis illustrates this point. Although a very small proportion of individual genes had low p-values in the *p53 *dataset, SAM-GS indicated larger proportions of gene sets with low p-values. This is because a valid gene-set analysis would take into account a tendency among multiple genes in a gene set. Thus, even if the association of each gene with the phenotype is only moderate, a collection of such genes can be indicated as a phenotype-associated gene set; genes in a gene set need not have the same degree and direction of association with the phenotype for the gene set to be identified as statistically significant by SAM-GS.

In addition to the leukemia-comparison analysis discussed above, which showed an advantage of SAM-GS over GSEA empirically through the consistency of the gene-set analysis results with the individual-gene analysis results, the other two DNA-microarray analyses (sex- and *p53*-comparison analyses) provided empirical biologic evidence supporting the advantage of SAM-GS over GSEA. Regarding the sex-comparison analysis, Subramanian et al. [3] specifically argue that they would not expect to find enrichment for bands on the X chromosome because most X-linked genes are randomly silenced in females and, therefore, are unlikely to show a male-female (gene-dose) difference. This argument has general merit; however, in the specific case of the chrXp22 gene set, it does not hold because, on the distal portion of the short arm of X, there is a cluster of genes that escape X-inactivation. Indeed, the top five genes of the chrXp22 gene set escape inactivation: two of the five are members of the X-inactivation-escape gene set whose FDR was ≤ 0.01 by both methods; and the other three have been reported to escape X-inactivation [6-8].

The differences in the results of the *p53*-comparison analysis illuminate biologically relevant performance differences between the two methods. It is appropriate to ask whether the 31 additional pathways identified by SAM-GS over GSEA are plausibly associated with the presence vs. absence of *p53*. Of the 31 gene sets, 11 actually involve *p53 *directly as a member. A further 6 gene sets directly involve the extrinsic and intrinsic apoptosis pathways [9], 3 involve the cell-cycle machinery, and 3 involve cytokines and/or JAK/STAT signaling [10]. Each of these 12 gene sets, then, is in a direct, well-established relationship with aspects of *p53 *signaling. Of the remaining 8 gene sets, 6 have plausible, if less well established, links with *p53*. In the Ck1 pathway, cdk5 phosphorylates *p53 *so the presence vs. absence of *p53 *is likely to modify profoundly the effectiveness of this pathway [11]. Ets1 (ets pathway) has been shown to be essential, in mouse embryonic stem cells, to maintain the ability to undergo UV-induced, *p53*-dependent apoptosis. Ets1, more broadly, may be necessary for *p53*-dependent gene transactivation [12]. Akt and *p53 *are, respectively, essential to the transduction of anti-apoptotic and pro-apoptotic pathways. There is an integrated negative feedback loop whereby *p53*-dependent down regulation of Akt promotes cell death but cell survival signals will recruit Akt, leading to activation of Mdm2 and the inhibition of *p53*-dependent apoptosis [13]. This may account, in part, for the association between the presence vs. absence of *p53 *and differences in the SA-TRKA receptor pathway. Proline oxidase is induced by *p53 *and mediates apoptosis via a calcineurin-dependent pathway [14]. Coproporphyrinogen oxidase (CPO) is a key compound of the MAP 00860 porphyrin/chlorophyll metabolism gene set. It catalyzes a rate-limiting step in heme biosynthesis and may contribute to mitochondrial redox balance. It has recently been shown to be regulated by *p53 *[15]. Finally, the Wnt and *p53 *pathways have also been shown to be linked via pro-apoptotic Dkk1, a wnt antagonist [16].

### SAM-GS

Regarding the form of *SAMGS *test statistic, ∑i=1|S|di2
 MathType@MTEF@5@5@+=feaafiart1ev1aaatCvAUfKttLearuWrP9MDH5MBPbIqV92AaeXatLxBI9gBaebbnrfifHhDYfgasaacH8akY=wiFfYdH8Gipec8Eeeu0xXdbba9frFj0=OqFfea0dXdd9vqai=hGuQ8kuc9pgc9s8qqaq=dirpe0xb9q8qiLsFr0=vr0=vr0dc8meaabaqaciaacaGaaeqabaqabeGadaaakeaadaaeWbqaaiabdsgaKnaaDaaaleaacqWGPbqAaeaacqaIYaGmaaaabaGaemyAaKMaeyypa0JaeGymaedabaGaeiiFaWNaem4uamLaeiiFaWhaniabggHiLdaaaa@3A2E@ is simply the *L*_*2*_-norm of the t-like-statistic vector *d *= (*d*_1_, *d*_2_, ⋯, *d*_|*s*|_), the length of the line segment joining the two phenotypes' mean gene-expression vectors of a gene set *S*. Our null hypothesis is that the mean vectors of expressions of genes in a gene set *S *do not differ by the phenotype of interest (i.e., this line-segment length is zero), a two-sample multivariate mean test in statistics. The classical multivariate statistics method for a two-sample mean test, Hotelling's *T*^2^, addresses this question, but it cannot be applied when |*S*| > *n*_1 _+ *n*_2 _- 2, where *n*_1 _and *n*_2 _are the sample sizes in the two groups defining the phenotype *D*. We would like to emphasize that this condition is often unmet in gene-set analyses of DNA microarray data. Dempster [16,17] introduced a test statistic for comparing highly multivariate samples of two groups, an alternative for Hotelling's *T*^2^, when the number of multivariate measurements is large, relative to the sample sizes. Using Dempster's test in the context of microarray data, a potential candidate for a test statistic to be used in Step 2 of SAM-GS, would be the weighted Dempster's (WD) statistic:

WD=∑i=1|S|di2/E^[∑i=1|S|di2],
 MathType@MTEF@5@5@+=feaafiart1ev1aqatCvAUfKttLearuWrP9MDH5MBPbIqV92AaeXatLxBI9gBaebbnrfifHhDYfgasaacH8akY=wiFfYdH8Gipec8Eeeu0xXdbba9frFj0=OqFfea0dXdd9vqai=hGuQ8kuc9pgc9s8qqaq=dirpe0xb9q8qiLsFr0=vr0=vr0dc8meaabaqaciaacaGaaeqabaqabeGadaaakeaacqWGxbWvcqWGebarcqGH9aqpdaaeWbqaaiabdsgaKnaaDaaaleaacqWGPbqAaeaacqaIYaGmaaaabaGaemyAaKMaeyypa0JaeGymaedabaGaeiiFaWNaem4uamLaeiiFaWhaniabggHiLdGccqGGVaWldaqiaaqaaiabdweafbGaayPadaGaei4waS1aaabCaeaacqWGKbazdaqhaaWcbaGaemyAaKgabaGaeGOmaidaaaqaaiabdMgaPjabg2da9iabigdaXaqaaiabcYha8jabdofatjabcYha8bqdcqGHris5aOGaeiyxa0LaeiilaWcaaa@512E@

where E^[∑i=1|S|di2]
 MathType@MTEF@5@5@+=feaafiart1ev1aqatCvAUfKttLearuWrP9MDH5MBPbIqV92AaeXatLxBI9gBaebbnrfifHhDYfgasaacH8akY=wiFfYdH8Gipec8Eeeu0xXdbba9frFj0=OqFfea0dXdd9vqai=hGuQ8kuc9pgc9s8qqaq=dirpe0xb9q8qiLsFr0=vr0=vr0dc8meaabaqaciaacaGaaeqabaqabeGadaaakeaadaqiaaqaaiabdweafbGaayPadaGaei4waS1aaabCaeaacqWGKbazdaqhaaWcbaGaemyAaKgabaGaeGOmaidaaaqaaiabdMgaPjabg2da9iabigdaXaqaaiabcYha8jabdofatjabcYha8bqdcqGHris5aOGaeiyxa0faaa@3E8E@ in the denominator is the average of (*n*_1 _+ *n*_2 _- 2) statistically-independent quantities that have the same mean and variance as the numerator ∑i=1|S|di2
 MathType@MTEF@5@5@+=feaafiart1ev1aaatCvAUfKttLearuWrP9MDH5MBPbIqV92AaeXatLxBI9gBaebbnrfifHhDYfgasaacH8akY=wiFfYdH8Gipec8Eeeu0xXdbba9frFj0=OqFfea0dXdd9vqai=hGuQ8kuc9pgc9s8qqaq=dirpe0xb9q8qiLsFr0=vr0=vr0dc8meaabaqaciaacaGaaeqabaqabeGadaaakeaadaaeWbqaaiabdsgaKnaaDaaaleaacqWGPbqAaeaacqaIYaGmaaaabaGaemyAaKMaeyypa0JaeGymaedabaGaeiiFaWNaem4uamLaeiiFaWhaniabggHiLdaaaa@3A2E@ under the null hypothesis, created by an orthonormal transformation of multivariate gene expressions in the set *S*. This test statistic seems to have the advantage of taking into account the multivariate structure of the gene expression measurements in a gene set by dividing the numerator, the *L*_*2*_-norm of the mean-vector difference, by its approximate expectation. However, since a permutation-based test is used, the denominator of WD statistic is unnecessary: as Dempster [18] stated, a permutation test based on the numerator only is equivalent to using the quotient. Given the computational simplicity and the use of permutation in SAM-GS, the *L*_*2*_-norm used in *SAMGS *is preferred over WD.

The *L*_*1*_-norm of *d *= (*d*_1_, *d*_2_, ⋯, *d*_|*s*|_), ∑i=1|S||di|
 MathType@MTEF@5@5@+=feaafiart1ev1aaatCvAUfKttLearuWrP9MDH5MBPbIqV92AaeXatLxBI9gBaebbnrfifHhDYfgasaacH8akY=wiFfYdH8Gipec8Eeeu0xXdbba9frFj0=OqFfea0dXdd9vqai=hGuQ8kuc9pgc9s8qqaq=dirpe0xb9q8qiLsFr0=vr0=vr0dc8meaabaqaciaacaGaaeqabaqabeGadaaakeaadaaeWbqaaiabcYha8jabdsgaKnaaBaaaleaacqWGPbqAaeqaaaqaaiabdMgaPjabg2da9iabigdaXaqaamaaemaabaGaem4uamfacaGLhWUaayjcSdaaniabggHiLdGccqGG8baFaaa@3C67@ can be considered, similar to Chung and Fraser [19] who proposed the *L*_*1*_-norm as an alternative to Dempster's use of the *L*_*2*_-norm. While one might expect the two norms to give similar performances overall, since the *L*_*1*_-norm would be less sensitive to extreme values than the *L*_*2*_-norm, the *L*_*1*_-norm may be less powerful in detecting a gene-set with a small number of genes being strongly associated with the phenotype. Test 2 simulation above confirmed this point: as the proportion of genes in a gene set that are correlated with the phenotype (|*r*| ≥ 0.6) becomes smaller than approximately 30%, the two norms performs differently and the *L*_*1*_-norm is less powerful in detecting the gene set being associated with the phenotype (data not shown).

To account for multiple comparisons (statistical testing of many hypotheses) when multiple gene sets are to be tested, SAM-GS takes the same approach as SAM, estimating a q-value, an upper limit for the FDR, for each gene set. The q-value of a gene set can be determined solely from the p-values of all gene sets tested [20]. The collection of p-values of all gene sets contains information, not only on the statistical significance of each gene for its association with the phenotype, but also on the proportion of gene sets that are not associated with the phenotype, the "null gene-set proportion." Note that the null gene-set proportion is determined by biology: the phenotype is either biologically associated or not associated with each gene set. However, the p-value is a function of sample sizes and noise levels in gene-expression measurements as well as the degree of underlying biological associations. Thus, even if a strong biologic association between a gene set and the phenotype exists, because of small sample sizes and/or high measurement noise levels (features of many DNA microarray observations and experiments), the p-value of the gene set can be large. This is another aspect of the *p53 *analysis discussed above, where many gene sets have low FDR estimates in spite of the fact that the p-values are not correspondingly low: this is due to an estimated small null gene-set proportion which lowers FDR estimates.

## Conclusion

In conclusion, GSEA is subject to some serious problems as a method for gene-set analysis, potentially leading to unnecessarily high false-positive and false-negative discovery rates. SAM-GS, based on the SAM t-like statistic, is proposed as an alternative gene-set analysis method that is statistically sound and has advantages, as illustrated in this paper, from both statistical and empirical biologic perspectives.

## Methods

### GSEA for gene-set analyses

A gene-set analysis for an *a priori *defined set of genes *S *in a total of *N *genes, on a DNA microarray is a test of the null hypothesis that the expression pattern of *S *is not associated with a phenotype of interest, *D*. To simplify the discussion, we will consider only a phenotype with two categories, {0, 1}: e.g. presence or absence of a disease. As biologists are often interested in testing multiple gene-sets {*S*_*1*_, ..., *S*_*k*_}, we will also consider a gene-set analysis for multiple gene-sets, following our discussion of an individual gene-set.

The revised version of GSEA by [3], for *an individual gene-set*, proceeds as follows.

### GSEA Steps

1) Compute the Pearson correlation (or another metric) between each of the *N *genes with a phenotype *D*, where the correlation or another metric of the *i*^th ^gene is denoted by *r*_*i*_.

2) Order the *N *genes by their correlation values from the maximum to the minimum (the ordered list is denoted by *L*).

3) Compute the Enrichment Score (ES): start with ES = 0; walk down the ranked list L, from the top rank (*i *= 1) to the last rank (*i *= *N*), increasing ES by |ri|/∑j∈S|rj|
 MathType@MTEF@5@5@+=feaafiart1ev1aaatCvAUfKttLearuWrP9MDH5MBPbIqV92AaeXatLxBI9gBaebbnrfifHhDYfgasaacH8akY=wiFfYdH8Gipec8Eeeu0xXdbba9frFj0=OqFfea0dXdd9vqai=hGuQ8kuc9pgc9s8qqaq=dirpe0xb9q8qiLsFr0=vr0=vr0dc8meaabaqaciaacaGaaeqabaqabeGadaaakeaacqGG8baFcqWGYbGCdaWgaaWcbaGaemyAaKgabeaakiabcYha8jabc+caVmaaqafabaGaeiiFaWNaemOCai3ccqWGQbGAkiabcYha8bWcbaGaemOAaOMaeyicI4Saem4uamfabeqdcqGHris5aaaa@3FA2@ if the *i*^th ^gene belongs to the gene set *S*, and decreasing ES by 1/(*N *- |*S*|) otherwise, where|*S*| is the number of genes in the set *S*.

4) Take the absolute value of the maximum deviation from zero of the ES values among the *N *genes as the test statistic for the gene set *S*.

5) Permute the labels of the phenotype *D *and repeat steps 1)- 4). Repeat until all (or a large number of) permutations are considered.

6) Statistical significance for the association of *S *and *D *is obtained by comparing the observed value of the test statistic from 3) and its permutation distribution from 5).

The initial version of GSEA proposed in Step [2] used 1/|*S*|, instead of |ri|/∑j∈S|rj|
 MathType@MTEF@5@5@+=feaafiart1ev1aaatCvAUfKttLearuWrP9MDH5MBPbIqV92AaeXatLxBI9gBaebbnrfifHhDYfgasaacH8akY=wiFfYdH8Gipec8Eeeu0xXdbba9frFj0=OqFfea0dXdd9vqai=hGuQ8kuc9pgc9s8qqaq=dirpe0xb9q8qiLsFr0=vr0=vr0dc8meaabaqaciaacaGaaeqabaqabeGadaaakeaacqGG8baFcqWGYbGCdaWgaaWcbaGaemyAaKgabeaakiabcYha8jabc+caVmaaqafabaGaeiiFaWNaemOCai3ccqWGQbGAkiabcYha8bWcbaGaemOAaOMaeyicI4Saem4uamfabeqdcqGHris5aaaa@3FA2@, for increasing the ES for each gene in *S*. The use of |ri|/∑j∈S|rj|
 MathType@MTEF@5@5@+=feaafiart1ev1aaatCvAUfKttLearuWrP9MDH5MBPbIqV92AaeXatLxBI9gBaebbnrfifHhDYfgasaacH8akY=wiFfYdH8Gipec8Eeeu0xXdbba9frFj0=OqFfea0dXdd9vqai=hGuQ8kuc9pgc9s8qqaq=dirpe0xb9q8qiLsFr0=vr0=vr0dc8meaabaqaciaacaGaaeqabaqabeGadaaakeaacqGG8baFcqWGYbGCdaWgaaWcbaGaemyAaKgabeaakiabcYha8jabc+caVmaaqafabaGaeiiFaWNaemOCai3ccqWGQbGAkiabcYha8bWcbaGaemOAaOMaeyicI4Saem4uamfabeqdcqGHris5aaaa@3FA2@, or more generally |ri|p/∑j∈S|rj|p
 MathType@MTEF@5@5@+=feaafiart1ev1aqatCvAUfKttLearuWrP9MDH5MBPbIqV92AaeXatLxBI9gBaebbnrfifHhDYfgasaacH8akY=wiFfYdH8Gipec8Eeeu0xXdbba9frFj0=OqFfea0dXdd9vqai=hGuQ8kuc9pgc9s8qqaq=dirpe0xb9q8qiLsFr0=vr0=vr0dc8meaabaqaciaacaGaaeqabaqabeGadaaakeaacqGG8baFcqWGYbGCdaWgaaWcbaGaemyAaKgabeaakiabcYha8naaCaaaleqabaGaemiCaahaaOGaei4la8YaaabuaeaacqGG8baFcqWGYbGCliabdQgaQPGaeiiFaWhaleaacqWGQbGAcqGHiiIZcqWGtbWuaeqaniabggHiLdGcdaahaaWcbeqaaiabdchaWbaaaaa@42E3@, was motivated by the need to reduce the ES values and the statistical significance of sets clustered near the middle of the ranked list (see Figure 1 and Table 1 in [3]). Although the modified version of GSEA was aimed at reducing the statistical significance of sets not exhibiting biologically relevant correlation with the phenotype, serious problems remain with GSEA as demonstrated here. To run GSEA, we used the Desktop application downloaded from [21], and the options specified in [3], that is, the Pearson correlation of the gene expressions with the phenotype to rank the genes, and weighted ES.

### The proposed method, SAM-GS

The main aim of analyzing *an individual gene-set *is to distinguish between the two biologic conditions (phenotype) based on multivariate measurements of the expression of genes in the gene set. GSEA tests a null hypothesis that rankings of the genes in a gene set according to an association measure with the phenotype categories (e.g., correlation) are randomly distributed over the rankings of all genes, using Kolmogorov-Smirnov statistic. SAM-GS, on the other hand, tests a null hypothesis that the mean vectors of expressions of genes in a gene set does not differ by the phenotype of interest.

Our proposed SAM-GS method is based on individual t-like statistics from SAM, addressing the small variability problem encountered in microarray data, i.e., reducing the statistical significance associated with genes with very little variation in their expressions. SAM-GS for *an individual gene-set *can be summarized in a few steps.

### SAM-GS Steps

1) For each of the *N *genes, calculate the statistic *d *as in SAM for an individual-gene analysis:

di=x¯1(i)−x¯2(i)s(i)+s0,
 MathType@MTEF@5@5@+=feaafiart1ev1aqatCvAUfKttLearuWrP9MDH5MBPbIqV92AaeXatLxBI9gBaebbnrfifHhDYfgasaacH8akY=wiFfYdH8Gipec8Eeeu0xXdbba9frFj0=OqFfea0dXdd9vqai=hGuQ8kuc9pgc9s8qqaq=dirpe0xb9q8qiLsFr0=vr0=vr0dc8meaabaqaciaacaGaaeqabaqabeGadaaakeaacqWGKbazdaWgaaWcbaGaemyAaKgabeaakiabg2da9maalaaabaWaa0aaaeaacqWG4baEaaWaaSbaaSqaaiabigdaXaqabaGccqGGOaakcqWGPbqAcqGGPaqkcqGHsisldaqdaaqaaiabdIha4baadaWgaaWcbaGaeGOmaidabeaakiabcIcaOiabdMgaPjabcMcaPaqaaiabdohaZjabcIcaOiabdMgaPjabcMcaPiabgUcaRiabdohaZnaaBaaaleaacqaIWaamaeqaaaaakiabcYcaSaaa@45DF@

where the 'gene-specific scatter' *s*(*i*) is a pooled standard deviation over the two groups of the phenotype, and *s*_0 _is a small positive constant that adjusts for the small variability encountered in microarray data [1].

2) Compute the *SAMGS *test statistic corresponding to set *S*:

SAMGS=∑i=1|S|di2
 MathType@MTEF@5@5@+=feaafiart1ev1aaatCvAUfKttLearuWrP9MDH5MBPbIqV92AaeXatLxBI9gBaebbnrfifHhDYfgasaacH8akY=wiFfYdH8Gipec8Eeeu0xXdbba9frFj0=OqFfea0dXdd9vqai=hGuQ8kuc9pgc9s8qqaq=dirpe0xb9q8qiLsFr0=vr0=vr0dc8meaabaqaciaacaGaaeqabaqabeGadaaakeaacqWGtbWucqWGbbqqcqWGnbqtcqWGhbWrcqWGtbWucqGH9aqpdaaeWbqaaiabdsgaKnaaDaaaleaacqWGPbqAaeaacqaIYaGmaaaabaGaemyAaKMaeyypa0JaeGymaedabaGaeiiFaWNaem4uamLaeiiFaWhaniabggHiLdaaaa@40D7@

3) Permute the labels of the phenotype *D *and repeat 1) and 2). Repeat until all (or a large number of) permutations are considered.

4) Statistical significance for the association of *S *and *D *is obtained by comparing the observed value of the *SAMGS *statistic from 2) and its permutation distribution from 3).

Note that SAM-GS initially measures the gene-expression difference across the binary phenotype in each gene *i *of the gene set *S *using *d*_*i*_, where the differences are standardized across the genes for their degrees of scatter with the denominators of *d*_*i*_'s, {*s*(*i*) + *s*_*0*_}. It then summarizes these standardized differences in all the genes in the gene set *S *by *SAMGS*. The analysis of multiple gene sets can be accommodated in SAM-GS by estimating false discovery rates (FDRs) from p-values of individual sets using the q-value method of [20].

### Gene-set simulation experiment

To illustrate the differences between SAM-GS and GSEA, we compared them on the simple requisite properties (a), (b), and (c), described in Results and Discussion, for any method designed to perform a gene-set analysis. We performed two tests using mouse-microarray data, simulating gene sets.

**Test 1**: Sample *n *genes by a simple random sampling as a hypothetical gene set from a group of genes with no or weak association with the phenotype, i.e., genes with |*r*| <*c*, where *c *is 0.01, 0.02, 0.05, 0.1, 0.2, 0.3, 0.4, or 0.5. Test the association of this *n*-gene set with the phenotype. Repeat 100 times to check property (a) for each value of *c*.

**Test 2**: Sample *n *genes by a stratified random sampling as a hypothetical gene set such that half of the genes in the set are moderately or strongly associated with the phenotype with |*r*| <*c*, and the other half with |*r*| <*c *where *c *is 0.4, 0.5, 0.6, 0.7, 0.8, or 0.9. Test the association of this *n*-gene set with the phenotype. Repeat 100 times to check property (b) for each value of *c*.

In Test 1, our simple random sampling from the no-or-weak association region creates gene sets that approximate to the null hypothesis such that their members are consistently not associated with the phenotype (e.g., they have a mixture of genes with correlations between -0.01 and 0.01 when *c *= 0.01) or are variably weakly associated, or not associated (e.g., correlations between -0.4 and 0.4, including many around zero, when *c *= 0.4). These gene sets should not be called significantly associated with the phenotype. In Test 2, as half of the genes in the gene set are moderately or strongly associated with the phenotype, these gene sets should be identified as significantly associated with the phenotype.

The two tests were performed based on the data from the mouse-microarray kidney transplant study. In this study, we compared two experimental groups of mouse kidney transplants: fully MHC mismatched allografts and MHC identical isografts. A more detailed description of the study is given in the Appendix. Briefly, in both groups, the kidneys have undergone the same surgical procedure of transplantation, but in addition the allograft develops the histologic lesions of rejection due to the immune response by the host, while the isograft does not develop these lesions due to an identical genetic background. We have studied a full timecourse between days 1 and 42 post transplant; the alloimmune response is fully developed at days 5–7, and the injury response in the isografts also peaks at days 5–6. To simplify the comparison between rejecting allografts and non-rejecting isografts, we have therefore selected the data from days 5 and 7 as the basis of this analysis. A total of 12 samples were analyzed: 3 samples each at day 5 and day 7 in allografts, 4 samples in day 5 isografts, and 2 samples in day 7 isografts. The microarray data were obtained by hybridizing mRNA to Affymetrix MOE 430 2.0 microarrays. These arrays contain 45,099 probe sets whose expression was reduced from the probe level to the gene level of 16,612 unique genes by a method described in the GSEA website, by taking the maximum probe set expression of each gene in each sample. We considered a gene set size *n *of 10, 30, 50, and 100. We ran the same gene-set simulation experiment using the three datasets (sex, *p53*, leukemia). Results and relevant discussions are given separately as an additional file [see Additional file 1].

### Gene-set analyses of three datasets with biologically defined gene sets

We compared the performance of the two methods, GSEA and SAM-GS, on the analyses of biologically defined gene sets using three microarray datasets considered in [3]: male vs. female lymphoblastoid cells; *p53 *wild-type vs. mutant cancer cell lines; and ALL vs. AML leukemia cells. The comparison used GSEA results for the three examples, downloaded from GSEA web-page, [21]. We used the datasets and gene-set subcatalogs C1 and C2 from the above web address to be exactly comparable with the GSEA paper [3]. The same datasets and subcatalogs were used for both GSEA and SAM-GS, only including gene sets with sizes between 5 and 500, following the GSEA paper [3].

We did not analyze the lung adenocarcinoma data of three studies (Boston, Michigan, and Stanford studies) in [3] as such an analysis is methodologically problematic: the Michigan study included only patients with stage I or III lung adenocarcinoma, whereas the Boston and Stanford studies did not restrict the stages; the binary phenotype of interest, death, was defined using censored survival data, where the length of follow-up to ascertain death varied appreciably both by patient and across studies (the median follow-up was 49.9, 29.5, and 17.5 months in the Boston, Michigan, and Stanford studies, respectively), leading to inconsistent ascertainment of the binary phenotype (death) across patients and studies (patients with a longer follow-up had a higher chance of being ascertained to have died); and no adjustment was applied to control for possible differences across the studies in treatment, tumor characteristics, and demographics of the patients.

## Abbreviations

Gene Set Enrichment Analysis (GSEA)

Significance Analysis of Microarray for Gene Sets (SAM-GS)

## Availability and requirements

Project name: Statistical methods for biomarker discovery based on high-dimensional gene/protein expression profiles

Project home page: 

Operating system(s): Microsoft Windows XP

Programming language: R 2.4.1 and Microsoft Excel 2000

## Authors' contributions

ID and YY developed the SAM-GS methodology and designed/conducted the methodological study. TM, GE, KF, GJ, and PH performed the mouse transplant microarray study and introduced the gene-set analysis problem to ID and YY, including the GSEA methodology. JP provided biological interpretations of the analysis results of three real-world datasets, in particular, the *p53 *related pathways. QL and AA contributed significantly to data analysis, refinement of SAM-GS, and programming. The manuscript was written primarily by ID, YY, and JP, and critically reviewed and revised by all authors. All authors read and approved the final manuscript.

## Appendix

### Mouse-microarray kidney transplant study

A histogram of Pearson correlation with the phenotype for 16,612 individual genes in the mouse-microarray kidney-transplant study is given separately as an additional file [see Additional file 2].

#### Mice

Male CBA/J (CBA) and C57Bl/6 (B6) were obtained from Jackson Laboratory (Bar Harbor, ME). Mice were maintained in the Health Sciences Laboratory Animal Services at the University of Alberta. All maintenance and experiments conformed to approved animal care protocols.

#### Transplants

Non-life-supporting renal transplants were performed across full MHC and non-MHC disparities as previously described [22] using wild-type CBA mice as donors and wild-type B6 or wild-type CBA as recipients. Hosts did not receive immunosuppression. Naïve kidneys of the appropriate strain and isografts served as controls. Kidneys were harvested on days 1, 2, 3, 4, 5, 7, 14, 21 and 42 post transplant as previously described [22], snap-frozen in liquid nitrogen and stored at -70°C until further analysis. CBA allografts rejecting in wild type hosts (B6) at days 1, 2, 3, 4, 5, 7, 14, 21 and 42 were designated allo.CBA D1–D42; isografts (CBA into CBA) named iso.CBA. Our mouse model of renal transplants across MHC disparities (CBA into B6), studied over the timecourse between days 1–42 simulates the development of the lesions of T cell mediated rejection that we observe in human biopsies. The isografts (CBA into CBA) serve as controls that have undergone the same surgical procedure without evoking an alloimmune response.

#### Microarrays

We performed microarray analysis on normal mouse kidneys (NCBA), in allo.CBA D1–D42, and isografts D1–21. RNA extraction, dsDNA and cRNA synthesis, hybridization to MOE430 2.0 oligonucleotide arrays (GeneChip, Affymetrix^®^), washing and staining were carried out according to [24] and as previously described[25]. Equal amounts of RNA from 3 mice (20–25 μg each) were pooled for each array. Data was normalized using RMA using GeneSpring™ software (Version 7.2, Silicon Genetics, CA, USA) as described previously [25].

## Supplementary Material

Additional file 1 Gene-set simulation experiment results with the sex, *p53*, and leukemia datasets. The results of the gene-set simulation experiments using the three datasets are given. Click here for file

Additional file 2 Histogram of Pearson correlation with the phenotype in the mouse-microarray kidney-transplant study. Histogram of Pearson correlation with the phenotype for 16,612 individual genes in the mouse-microarray kidney-transplant study. Click here for file
