# Supplementary material for: Improving gene set analysis of microarray data by SAM-GS
Source: BMC Bioinformatics. 2007 Jul 5;8:242. doi: 10.1186/1471-2105-8-242 (PMC1931607; doi:10.1186/1471-2105-8-242)
Supplement: Additional file 1 — Gene-set simulation experiment results with the sex, p53, and leukemia datasets. The results of the gene-set simulation experiments using the three datasets are given. [file 1471-2105-8-242-S1.pdf]

Gene-set simulation experiment results with the sex, *p53*, and leukemia datasets

We present here the results of the gene-set simulation experiment, applied to the three datasets of sex, *p53*, and leukemia comparisons. The distribution of the Pearson correlation with the phenotype across all genes is displayed for each dataset in Figures A1a, A1b, and A1c for the sex, *p53*, and leukemia datasets, respectively.

Regarding Test 1, GSEA incorrectly calls null gene sets (gene sets with genes uncorrelated with the phenotype) as statistically significant in all datasets: the more uncorrelated gene sets' genes are with the phenotype, the more frequently GSEA identifies the gene sets as being associated with the phenotype (Tables A1a, A1b, A1c). Not only the absolute values of correlation in the gene set, but also the distribution of correlation across all genes, influences the performance of GSEA, however. Specifically, GSEA calls gene sets whose genes are "clustered" along the correlation (or other association measure) axis. In the case of the mouse dataset, gene sets with their members from $|r|<0.1$, 0.2, 0.3, 0.4, or 0.5 had such clustering of genes along the correlation axis, because the distribution of correlation across all genes ranged widely for the whole range of correlation (see the histogram shown in Appendix) with an Inter Quartile Range (IQR) of 0.94. In the other three datasets, however, because the distribution of correlation across all genes was narrower (see Figures A1a, A1b, and A1c) with IQRs of 0.24, 0.22, and 0.58 for the sex, *p53*, and leukemia datasets, respectively, gene sets with their members from $|r|<0.1$, 0.2, 0.3, 0.4, or 0.5 did not have the clustering, unless $|r|<0.1$. In other words, GSEA showed the same pattern in the other three datasets as the mouse dataset of incorrectly calling null gene sets as statistically significant, even for the gene

sets whose members had smaller values of correlation with the phenotype (i.e., $|r| < 0.01$, 0.02, 0.05, and 0.1).

Note that Pearson correlation alone does not necessarily measure the degree of association with the phenotype. For example, in the leukemia dataset, two thirds of the genes with $|r|<0.5$ had FDR of 0.01 or lower. Thus, Tables 1, A1a, A1b, and A1c give the percentages of genes with FDR≤0.01 among genes in each of $|r|<0.01$, 0.02, 0.05, 0.1, 0.2, 0.3, 0.4, and 0.5 in each dataset, so that the degree of associations for individual genes is shown. In the case of the leukemia dataset, these percentages of genes with FDR≤0.01 supports that it is reasonable to call some of the gene sets with their members from $|r|<0.3$, 0.4, and 0.5 significant.

Another point relevant in interpreting our simulated gene-set analysis results is the symmetry between up-regulated and down-regulated genes. Recall that, in GSEA, up-regulated and down-regulated genes contribute to the test statistic separately, even if they are associated with the phenotype to an equal degree. Therefore, under a symmetry around zero correlation (or any other association measured used), GSEA is less likely to identify gene sets as being significantly associated with the phenotype, due to the separated counting of up-regulated and down-regulated genes in its test statistic. This resulted in, for example, the difference of 0% vs. 39% between the sex and *p53* datasets in the proportion of null gene sets of size 10 randomly generated from $|r|<0.01$ that were identified by GSEA with a p-value ≤ 0.05. The sex dataset was more symmetric around zero correlation (i.e., more balance in the numbers of up-regulated and down-regulated

genes) than the right-skewed *p53* dataset as seen in Figures A1a and A1b. Thus, there were more null gene sets of size 10 from $|r|<0.01$ in the *p53* dataset than in the sex dataset, whose associations with the phenotypes were identified by GSEA as significant with a p-value ≤ 0.05, even though the percentage of all genes in the region of $|r|<0.01$ was 4% in both datasets.

Regarding Test 2, GSEA shows appreciably lower power than SAM-GS for detecting non-null gene sets with half of their genes with moderate/high correlation with the phenotype in all four datasets (Tables A2a, A2b, and A2c). The distribution of correlation across all genes influences the performance of GSEA: thus, the degree of discrepancy between GSEA and SAM-GS differ across the four datasets.

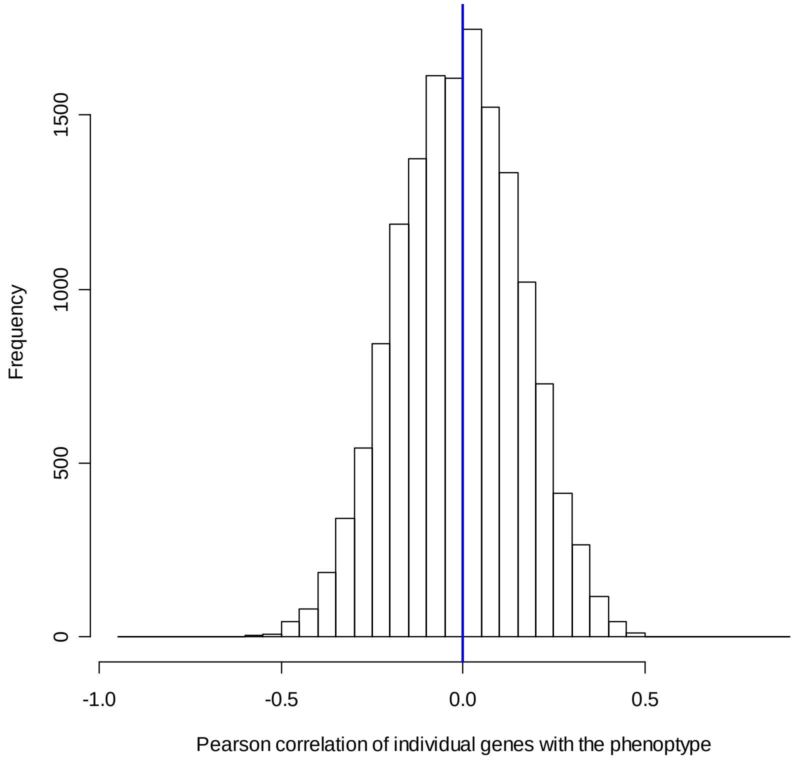

Figure A1a. Histogram of Pearson correlation with the phenotype for 15,056 individual genes in the sex dataset.

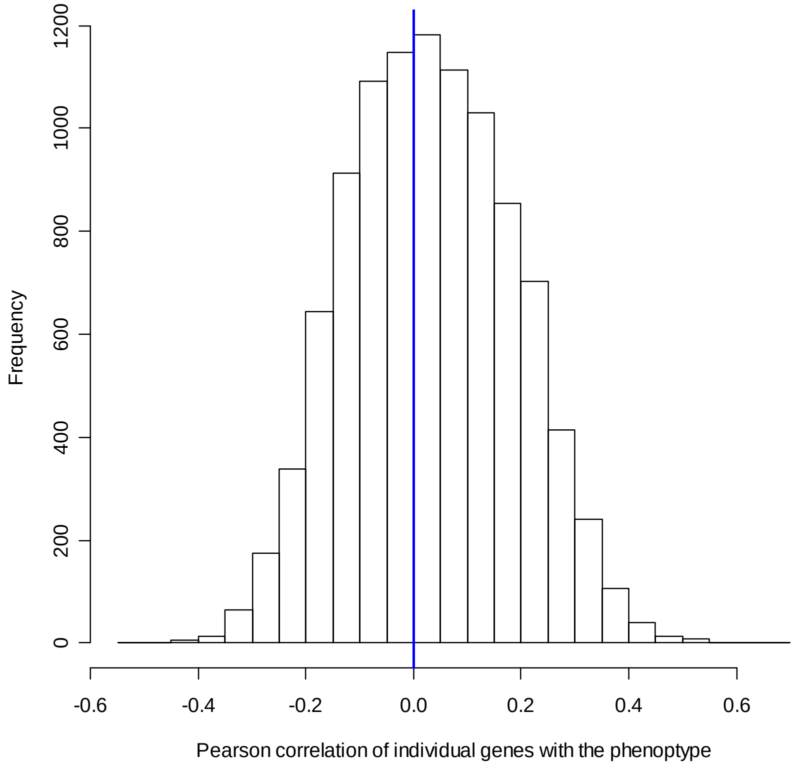

Figure A1b. Histogram of Pearson correlation with the phenotype for 10,100 individual genes in the *p53* dataset.

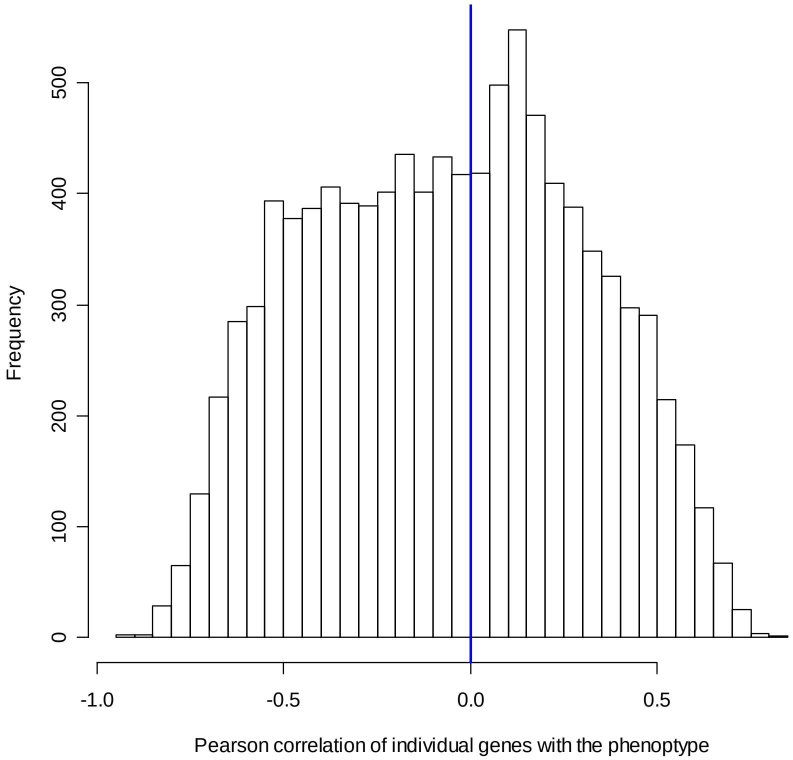

Figure A1c. Histogram of Pearson correlation with the phenotype for 10,056 individual genes in the leukemia dataset.

Table A1a. Performance of GSEA and SAM-GS on Test 1. Proportions of randomly generated null gene sets that are identified by each method to be associated with the phenotype (p-value ≤ 0.05) in the sex dataset.

**Correlation range from which gene-set members were selected (% of individual genes in the range) [% of individual genes in the range with FDR≤0.01]**	**Methods**	**Set Size**			
		**10**	**30**	**50**	**100**
$\|r\|<.01$ (4% of all genes are in the range) [0% with FDR≤.01]	GSEA	0%	100%	100%	100%
	SAM-GS	0%	0%	0%	0%
$\|r\|<.02$ (9% of all genes are in the range) [0% with FDR≤.01]	GSEA	0%	100%	100%	100%
	SAM-GS	0%	0%	0%	0%
$\|r\|<.05$ (22% of all genes are in the range) [0% with FDR≤.01]	GSEA	0%	100%	100%	100%
	SAM-GS	0%	0%	0%	0%
$\|r\|<.1$ (43% of all genes are in the range) [0% with FDR≤.01]	GSEA	0%	0%	7%	100%
	SAM-GS	0%	0%	0%	0%
$\|r\|<.2$ (76% of all genes are in the range)* [0% with FDR≤.01]	GSEA	2%	0%	0%	0%
	SAM-GS	0%	0%	0%	0%
$\|r\|<.3$ (93% of all genes are in the range)* [0% with FDR≤.01]	GSEA	3%	1%	1%	0%
	SAM-GS	0%	0%	0%	0%
$\|r\|<.4$ (98% of all genes are in the range)* [0% with FDR≤.01]	GSEA	4%	4%	7%	2%
	SAM-GS	0%	0%	0%	0%
$\|r\|<.5$ (99% of all genes are in the range)* [0.01% with FDR≤.01]	GSEA	4%	1%	4%	6%
	SAM-GS	0%	0%	0%	0%

*Due to the large percentage of genes in this correlation range, gene-sets generated from this range do not show clustering along the correlation axis. GSEA does not identify these gene sets as being significantly associated with the phenotype because of this lack of clustering.

Table A2a. Performance of GSEA and SAM-GS on Test 2. Proportions of randomly generated non-null gene sets that are identified by each method to be associated with the phenotype (p-value ≤ 0.05) in the sex dataset. The dash (-) represents situations where sampling was not possible because of lack of enough genes in the sampling region.

**Pearson correlation of genes in the gene set with the phenotype**	**Methods**	**Set Size**			
		**10**	**30**	**50**	**100**
Half of genes with $\|r\| \geq .4$ [&], the other half with $\|r\| < .4$	GSEA	46%	95%	100%	100%
	SAM-GS	100%	100%	100%	100%
Half of genes with $\|r\| \geq .5$ [&], the other half with $\|r\| < .5$	GSEA	51%	98%	100%	-
	SAM-GS	100%	100%	100%	-
Half of genes with $\|r\| \geq .6$ [&], the other half with $\|r\| < .6$	GSEA	26%	100%	-	-
	SAM-GS	100%	100%	-	-
Half of genes with $\|r\| \geq .7$ [&], the other half with $\|r\| < .7$	GSEA	64%	-	-	-
	SAM-GS	100%	-	-	-
Half of genes with $\|r\| \geq .8$ [&], the other half with $\|r\| < .8$	GSEA	57%	-	-	-
	SAM-GS	100%	-	-	-
Half of genes with $\|r\| \geq .9$, the other half with $\|r\| < .9$	GSEA	-	-	-	-
	SAM-GS	-	-	-	-

[&] Due to the small percentage of genes in this correlation range, half of the genes in the generated gene-sets show clustering along the correlation axis, leading to some gene sets identified by GSEA as being significantly associated with the phenotype.

Table A1b. Performance of GSEA and SAM-GS on Test 1. Proportions of randomly generated null gene sets that are identified by each method to be associated with the phenotype (p-value ≤ 0.05) in the *p53* dataset.

**Correlation range from which gene-set members were selected<br>(% of individual genes in the range)<br>[% of individual genes in the range with FDR≤0.01]**	**Methods**	**Set Size**			
		**10**	**30**	**50**	**100**
$\|r\|<.01$<br>(4% of all genes are in the range)<br>[0% with FDR≤.01]	GSEA	39%	100%	100%	100%
	SAM-GS	0%	0%	0%	0%
$\|r\|<.02$<br>(9% of all genes are in the range)<br>[0% with FDR≤.01]	GSEA	10%	100%	100%	100%
	SAM-GS	0%	0%	0%	0%
$\|r\|<.05$<br>(23% of all genes are in the range)<br>[0% with FDR≤.01]	GSEA	0%	100%	100%	100%
	SAM-GS	0%	0%	0%	0%
$\|r\|<.1$<br>(44% of all genes are in the range)<br>[0% with FDR≤.01]	GSEA	0%	29%	100%	100%
	SAM-GS	0%	0%	0%	0%
$\|r\|<.2$<br>(79% of all genes are in the range) *<br>[0% with FDR≤.01]	GSEA	4%	1%	1%	3%
	SAM-GS	0%	0%	0%	0%
$\|r\|<.3$<br>(95% of all genes are in the range) *<br>[0.01% with FDR≤.01]	GSEA	3%	2%	3%	0%
	SAM-GS	0%	0%	0%	0%
$\|r\|<.4$<br>(99% of all genes are in the range) *<br>[0.01%‖ with FDR≤.01]	GSEA	4%	6%	5%	4%
	SAM-GS	1%	4%	6%	3%
$\|r\|<.5$<br>(99.9% are in the range) *<br>[0.95% with FDR≤.01]	GSEA	5%	5%	8%	6%
	SAM-GS	9%	11%	19%	13%

*Due to the large percentage of genes in this correlation range, gene-sets generated from this range do not show clustering along the correlation axis. GSEA does not identify these gene sets as being significantly associated with the phenotype because of this lack of clustering.

Table A2b. Performance of GSEA and SAM-GS on Test 2. Proportions of randomly generated non-null gene sets that are identified by each method to be associated with the phenotype (p-value ≤ 0.05) in the *p53* dataset. The dash (-) represents situations where sampling was not possible because of lack of enough genes in the sampling region.

**Pearson correlation of genes in the gene set with the phenotype**	**Methods**	**Set Size**			
		**10**	**30**	**50**	**100**
Half of genes with $\lvert r \rvert \geq .4$ [&], the other half with $\lvert r \rvert < .4$	GSEA	89%	100%	100%	100%
	SAM-GS	100%	100%	100%	100%
Half of genes with $\lvert r \rvert \geq .5$ [&], the other half with $\lvert r \rvert < .5$	GSEA	100%	-	-	-
	SAM-GS	100%	-	-	-
Half of genes with $\lvert r \rvert \geq .6$, the other half with $\lvert r \rvert < .6$	GSEA	-	-	-	-
	SAM-GS	-	-	-	-
Half of genes with $\lvert r \rvert \geq .7$, the other half with $\lvert r \rvert < .7$	GSEA	-	-	-	-
	SAM-GS	-	-	-	-
Half of genes with $\lvert r \rvert \geq .8$, the other half with $\lvert r \rvert < .8$	GSEA	-	-	-	-
	SAM-GS	-	-	-	-
Half of genes with $\lvert r \rvert \geq .9$, the other half with $\lvert r \rvert < .9$	GSEA	-	-	-	-
	SAM-GS	-	-	-	-

[&]Due to the small percentage of genes in this correlation range, half of the genes in the generated gene-sets show clustering along the correlation axis, leading to some gene sets identified by GSEA as being significantly associated with the phenotype.

Table A1c. Performance of GSEA and SAM-GS on Test 1. Proportions of randomly generated null gene sets that are identified by each method to be associated with the phenotype (p-value ≤ 0.05) in the leukemia dataset

**Correlation range from which gene-set members were selected (% of individual genes in the range) [% of individual genes in the range with FDR≤0.01]**	**Methods**	**Set Size**			
		**10**	**30**	**50**	**100**
$\|r\|<.01$ (1.8% of all genes are in the range) [0% with FDR≤.01]	GSEA	46%	100%	100%	100%
	SAM-GS	0%	0%	0%	0%
$\|r\|<.02$ (3.3% of all genes are in the range) [0% with FDR≤.01]	GSEA	18%	100%	100%	100%
	SAM-GS	0%	0%	0%	0%
$\|r\|<.05$ (8.3% of all genes are in the range) [0% with FDR≤.01]	GSEA	5%	100%	100%	100%
	SAM-GS	0%	0%	0%	0%
$\|r\|<.1$ (18% of all genes are in the range) [0% with FDR≤.01]	GSEA	0%	100%	100%	100%
	SAM-GS	0%	0%	0%	0%
$\|r\|<.2$ (36% of all genes are in the range) [.33% with FDR≤.01]	GSEA	0%	99%	100%	100%
	SAM-GS	0%	0%	0%	0%
$\|r\|<0.3$ (52% of all genes are in the range)* [2% with FDR≤0.01]#	GSEA	1%	1%	80%	100%
	SAM-GS	17%	26%	35%	52%
$\|r\|<0.4$ (66% of all genes are in the range)* [46% with FDR≤0.01]#	GSEA	3%	1%	2%	7%
	SAM-GS	76%	99%	100%	100%
$\|r\|<0.5$ (80% of all genes are in the range)* [63% with FDR≤0.01]#	GSEA	6%	0%	4%	1%
	SAM-GS	94%	100%	100%	100%

*Due to the large percentage of genes in this correlation range, gene-sets generated from this range do not show clustering along the correlation axis. GSEA does not identify these gene sets as being significantly associated with the phenotype because of this lack of clustering.

#Due to the appreciable non-zero percentage of genes in this correlation range that are significantly associated with the phenotype, gene-sets generated from this range are not necessarily null gene sets. SAM-GS identifies such non-null gene sets as being significantly associated with the phenotype.

Table A2c. Performance of GSEA and SAM-GS on Test 2. Proportions of randomly generated non-null gene sets that are identified by each method to be associated with the phenotype (p-value ≤ 0.05) in the leukemia dataset. The dash (-) represents situations where sampling was not possible because of lack of enough genes in the sampling region

**Pearson correlation of genes in the gene set with the phenotype**	**Methods**	**Set Size**			
		**10**	**30**	**50**	**100**
Half of genes with $\|r\| \geq .4$ [&], the other half with $\|r\| < .4$	GSEA	11%	14%	31%	31%
	SAM-GS	100%	100%	100%	100%
Half of genes with $\|r\| \geq .5$ [&], the other half with $\|r\| < .5$	GSEA	17%	44%	75%	94%
	SAM-GS	100%	100%	100%	100%
Half of genes with $\|r\| \geq .6$ [&], the other half with $\|r\| < .6$	GSEA	42%	90%	91%	100%
	SAM-GS	100%	100%	100%	100%
Half of genes with $\|r\| \geq .7$ [&], the other half with $\|r\| < .7$	GSEA	81%	100%	100%	100%
	SAM-GS	100%	100%	100%	100%
Half of genes with $\|r\| \geq .8$ [&], the other half with $\|r\| < .8$	GSEA	99%	100%	100%	-
	SAM-GS	100%	100%	100%	-
Half of genes with $\|r\| \geq .9$, the other half with $\|r\| < .9$	GSEA	-	-	-	-
	SAM-GS	-	-	-	-

[&]Due to the small percentage of genes in this correlation range, half of the genes in the generated gene-sets show clustering along the correlation axis, leading to some gene sets identified by GSEA as being significantly associated with the phenotype.
