# Supplementary material for: Improving gene set analysis of microarray data by SAM-GS
Source: BMC Bioinformatics. 2007 Jul 5;8:242. doi: 10.1186/1471-2105-8-242 (PMC1931607; doi:10.1186/1471-2105-8-242)

Histogram of Pearson correlation with the phenotype for 16,612 individual genes in the mouse-microarray kidney-transplant study

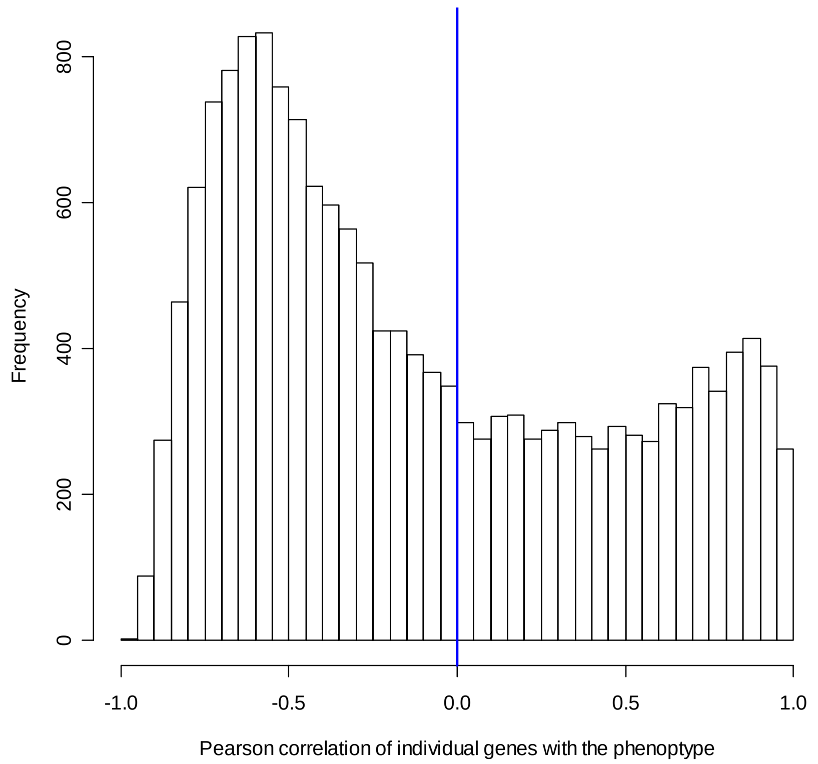

Supplement: Additional file 2 — Histogram of Pearson correlation with the phenotype in the mouse-microarray kidney-transplant study. Histogram of Pearson correlation with the phenotype for 16,612 individual genes in the mouse-microarray kidney-transplant study. [file 1471-2105-8-242-S2.pdf]
